# SARS-CoV-2 spike-reactive naïve B cells and pre-existing memory B cells contribute to antibody responses in unexposed individuals after vaccination

**DOI:** 10.3389/fimmu.2024.1355949

**Published:** 2024-02-14

**Authors:** Shishan Teng, Yabin Hu, You Wang, Yinggen Tang, Qian Wu, Xingyu Zheng, Rui Lu, Dong Pan, Fen Liu, Tianyi Xie, Chanfeng Wu, Yi-Ping Li, Wenpei Liu, Xiaowang Qu

**Affiliations:** ^1^ School of Public Health & School of Basic Medicine Sciences, Hengyang Medical School & Ministry of Education Key Laboratory of Rare Pediatric Diseases, University of South China, Hengyang, China; ^2^ Translational Medicine Institute, The First People’s Hospital of Chenzhou, Hengyang Medical School, University of South China, Chenzhou, China; ^3^ Institute of Human Virology, Zhongshan School of Medicine, and Key Laboratory of Tropical Disease Control of the Ministry of Education, Sun Yat-sen University, Guangzhou, China

**Keywords:** SARS-CoV-2, naïve B cell, preexisting memory B cell, vaccination, monoclonal antibody

## Abstract

**Introduction:**

Since December 2019, the emergence of severe acute respiratory syndrome coronavirus 2 (SARS-CoV-2) causing coronavirus disease 2019 (COVID-19) has presented considerable public health challenges. Multiple vaccines have been used to induce neutralizing antibodies (nAbs) and memory B-cell responses against the viral spike (S) glycoprotein, and many essential epitopes have been defined. Previous reports have identified severe acute respiratory syndrome coronavirus 2 (SARS-CoV-2) spike-reactive naïve B cells and preexisting memory B cells in unexposed individuals. However, the role of these spike-reactive B cells in vaccine-induced immunity remains unknown.

**Methods:**

To elucidate the characteristics of preexisting SARS-CoV-2 S-reactive B cells as well as their maturation after antigen encounter, we assessed the relationship of spike-reactive B cells before and after vaccination in unexposed human individuals. We further characterized the sequence identity, targeting domain, broad-spectrum binding activity and neutralizing activity of these SARS-CoV-2 S-reactive B cells by isolating monoclonal antibodies (mAbs) from these B cells.

**Results:**

The frequencies of both spike-reactive naïve B cells and preexisting memory B cells before vaccination correlated with the frequencies of spike-reactive memory B cells after vaccination. Isolated mAbs from spike-reactive naïve B cells before vaccination had fewer somatic hypermutations (SHMs) than mAbs isolated from spike-reactive memory B cells before and after vaccination, but bound SARS-CoV-2 spike *in vitro*. Intriguingly, these germline-like mAbs possessed broad binding profiles for SARS-CoV-2 and its variants, although with low or no neutralizing capacity. According to tracking of the evolution of IGHV4-4/IGKV3-20 lineage antibodies from a single donor, the lineage underwent SHMs and developed increased binding activity after vaccination.

**Discussion:**

Our findings suggest that spike-reactive naïve B cells can be expanded and matured by vaccination and cocontribute to vaccine-elicited antibody responses with preexisting memory B cells. Selectively and precisely targeting spike-reactive B cells by rational antigen design may provide a novel strategy for next-generation SARS-CoV-2 vaccine development.

## Introduction

Since December 2019, the emergence of severe acute respiratory syndrome coronavirus 2 (SARS-CoV-2) and, more recently, its novel variants causing coronavirus disease 2019 (COVID-19) has presented considerable challenges for public health (1). Multiple vaccines have been used, with the major goal of inducing the production of neutralizing antibodies (nAbs) and memory B cells to protect against SARS-CoV-2 infection.

The entry of SARS-CoV-2 into host cells is mediated by the viral spike (S) glycoprotein, the only known target for nAbs, which is composed of the S1 subunit encompassing an N-terminal domain (NTD) and the receptor-binding domain (RBD) responsible for receptor binding and the S2 subunit that is responsible for fusion of the viral and cellular membranes (2). Thus, most of the SARS-CoV-2 vaccines currently in use aim to induce an antibody response against the S protein. Many essential epitopes on S have been defined by using nAbs identified in early studies. The immunodominant S1 domain is the target of the most potent nAbs, and these S1-specific nAbs typically weakly cross-react with other human coronaviruses (HCoVs) and have not undergone extensive somatic hypermutation (SHM), suggesting that they originate from *de novo* activation of naïve B cell (3–7). However, nAbs targeting the more conserved S2 domain tend to be more broadly cross-reactive with coronaviruses (CoVs), suggesting that they may arise from the reactivation of preexisting memory B cells formed during endemic HCoV infection (8, 9).

Activation of naïve B cells usually occurs during initial antigen exposure. The sequence of membrane-bound B-cell receptors (BCRs) on naïve B cells determines their antigenic specificity (10). The potential BCR rearrangements of the human naïve B-cell repertoire enable these cells to recognize any essential antigen. Upon recognition, naïve B cells are activated and undergo SHM and subsequent differentiation to achieve affinity maturation (11–13). The frequency and intrinsic binding affinity of BCRs in the human naïve B-cell repertoire that recognize specific antigens determine their activation upon antigenic encounter (14, 15). The initial activation of the naïve repertoire often coincides with the eventual generation of protective antibodies and memory immunity (16, 17).

The existence of naïve B cells recognizing specific antigens in unexposed humans has been observed. Particularly for human immunodeficiency virus (HIV), the existence of germline precursors for broadly neutralizing antibodies (bnAbs) has been demonstrated, and germline-targeted immunogens with sufficient affinity for these precursors have been designed, offering innovative strategies for HIV vaccine development (18–21). For SARS-CoV-2, the presence of RBD-specific naïve B-cell precursors has been reported across the seronegative human population (22). Moreover, nAbs binding at the RBD-ACE2 interface were identified by screening human naïve antibody gene libraries collected prior to the emergence of SARS-CoV-2 (23, 24). In addition, a class of heavy chain immunoglobulin V gene (IGHV)1-69 and kappa chain immunoglobulin V gene (IGKV)3-11 germline-like antibodies have also been identified in unexposed individuals, which target nonneutralizing epitopes on the S2 subunit (25). These findings suggest the availability of SARS-CoV-2-specific B-cell precursors and their potential implications in the design of vaccines. It has also been found in some studies that preexisting immunity recognizing SARS-CoV-2 in uninfected individuals may come from the recall of HCoV-elicited cross-reactive memory B cells (9, 26–28).

Elucidating the characteristics of preexisting SARS-CoV-2 S-reactive B cells as well as their maturation after antigen encounter is therefore highly relevant for both understanding humoral immunity against SARS-CoV-2 and improving vaccines. Here, we characterized SARS-CoV-2 S-reactive B cells in the peripheral blood of an unexposed population before and after vaccination. We found that both S-reactive naïve B cells and preexisting memory B cells before vaccination cocontributed to antibody responses after vaccination. A series of monoclonal antibodies (mAbs) were isolated from these naïve B cells, which mainly target the S2 subunit and exhibit broad binding activities to SARS-CoV-2 and its variants. Tracking the evolution of an IGHV4-4/IGKV3-20 lineage revealed that these antibodies underwent SHMs and developed increased binding activity after vaccination. These findings provide novel strategy for the design of next-generation SARS-CoV-2 vaccines based on antigen-reactive naïve B-cell precursors in humans.

## Results

### SARS-CoV-2 S-reactive naïve and memory B cells cocontribute to humoral immunity after vaccination

**Figure 1:**
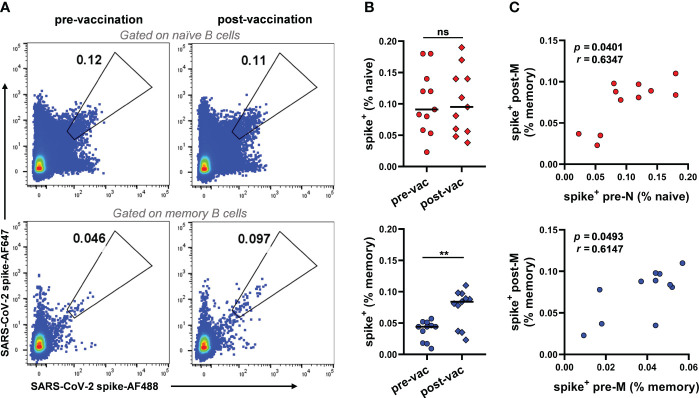
Frequencies of SARS-CoV-2 S-reactive B cells in unexposed individuals pre- and post-vaccination **(A)** Representative FACS plot of S-reactive B cells for naïve (CD3^-^CD19^+^CD27^-^IgD^+^IgG^-^) and memory (CD3^-^CD19^+^CD27^+^IgD^-^IgG^+^) B cells from donor V032 pre-and post-vaccination, respectively. **(B)** Frequencies of S-reactive B cells among naïve (top panel) and memory B cells (bottom panel) from each donor (n=11). Data are presented as the mean percent values. The difference between the pre- and post-vaccination groups was evaluated by a Wilcoxon matched-pairs signed rank test. Pre-vac, pre-vaccination, post-vac, post-vaccination. **p< 0.01, *p*< 0.05 was considered to indicate a two-tailed significant difference, ns, not significant. **(C)** The correlation of frequencies of S-reactive memory B cells post-vaccination and frequencies of S-reactive naïve (top panel) and memory B cells (bottom panel) pre-vaccination (n=11). Pre-N, pre-naïve, pre-M, pre-memory. The correlation between the two groups was evaluated by Spearman correlation analysis. *p<* 0.05 was considered to indicate a two-tailed significant difference.

To investigate the baseline frequency of SARS-CoV-2 S-reactive B cells prior to any SARS-CoV-2 S antigen encounter and the role of these cells in the vaccination-induced anti-SARS-CoV-2 humoral immune response, we sampled peripheral blood mononuclear cells (PBMCs) from 11 SARS-CoV-2-uninfected donors prior to vaccination and matched PBMCs two weeks after the second vaccine dose (**Table S1**). S-reactive naïve B cells (CD3^-^CD19^+^CD27^-^IgD^+^IgG^-^) and memory B cells (CD3^-^CD19^+^CD27^+^IgD^-^IgG^+^) were assessed with two fluorescence-labelled SARS-CoV-2 S probes in each donor (**Figure 1A**, **Figure S1**). SARS-CoV-2-reactive B cells were found in both naïve and classical memory B-cell subsets prior to vaccination, with median frequencies of 0.091% and 0.044%, respectively (**Figure 1B**). Although different fluorescent probes were used, consistent frequencies of SARS-CoV-2-reactive B cells in unexposed individuals were identified in a recent study (25) (0.11% of naïve and 0.049% of classical memory B cells). After the second vaccination dose, the frequency of SARS-CoV-2-reactive memory B cells appeared to double (median from 0.044% to 0.083%), while the frequency of SARS-CoV-2-reactive naïve B cells remained unaltered (from 0.091% to 0.095%, **Figure 1B**). The amplification of SARS-CoV-2-reactive memory B cells suggests the activation of humoral immunity induced by vaccination. To understand the potential origins of the amplification of SARS-CoV-2 reactive memory B cells post-vaccination (hereafter post-M), we performed a correlation analysis of the frequency of these cells with prevaccination SARS-CoV-2-reactive naïve (pre-N) and memory B cells (pre-M). The results showed that the frequency of post-M cells was positively correlated with the frequency of both pre-N and pre-M cells, suggesting that both SARS-CoV-2-reactive naïve and preexisting memory B cells may cocontribute to the development of anti-SARS-CoV-2 humoral immunity after vaccination (**Figure 1C**). This result is in line with recent studies on serum and mAbs from unexposed healthy donors and COVID-19 convalescents, suggesting that the antibody response against SARS-CoV-2 could stem from the naïve B-cell repertoire and cross-reactive memory B cells induced by prior infection with endemic HCoVs (9, 22, 25, 29).

### Characterization of SARS-CoV-2-reactive B cell-derived mAbs

**Figure 2:**
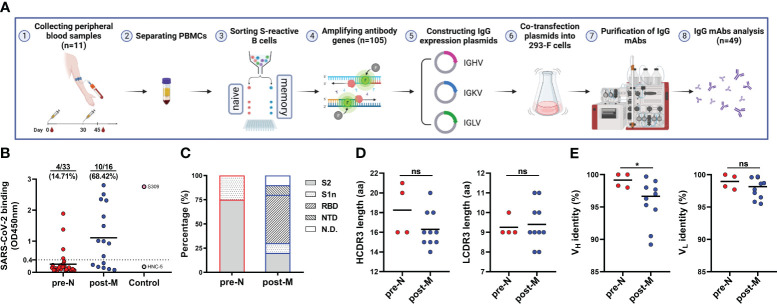
Isolation and characterization of SARS-CoV-2 S-reactive B cell-derived mAbs **(A)** Schematic diagram of the workflow of IgG mAb isolation. **(B)** mAb binding to SARS-CoV-2 S. Data are presented as the mean OD450 nm values. The numbers and percentages above indicate the proportion of SARS-CoV-2 S-binding mAbs. The cut-off value was defined as OD450 nm = 0.4, and mAbs with an OD450 nm > 0.4 were considered positive for binding. **(C)** Distribution of mAbs targeting domains in the S of SARS-CoV-2. S2, S2 subunit. S1n, S1 non-RBD/NTD domain. RBD, RBD. NTD, NTD. N.D., not detected. **(D)** HCDR3 and LCDR3 amino acid (aa) lengths of SARS-CoV-2 S-binding mAbs. Data are presented as the mean values. **(E)** Identity (%) to inferred germline gene sequences of V_H_ and V_L_ of SARS-CoV-2 S-binding mAbs. Data are presented as the mean percentages. For D and E, the difference between the two groups for CDR3 length and identity to the germline were evaluated by unpaired *t* test and Mann−Whitney test, respectively. *p< 0.05. *p<* 0.05 was considered to indicate a two-tailed significant difference, ns, not significant.

To understand naturally existing SARS-CoV-2-reactive B cells in more detail, we sorted single SARS-CoV-2-reactive pre-N, pre-M, and post-M cells from 9 of the 11 donors to amplify their antibody genes. We acquired 105 heavy chain (HC) and paired light chain (LC) sequences by reverse transcription-polymerase chain reaction (**Figure 2A**). Of the 105 pairs of sequences, 48, 20, and 37 were derived from pre-N, pre-M, and post-M cells, respectively. Sequence analysis showed noticeable enrichment of the IGHV4-34 (22.9% of total) and IGHV3-30 (20.8%) genes in pre-N cells, with both genes present in 5 of the 9 donors. IGHV3-30 (25.0%) was also enriched in pre-M cells (**Figure S2A**). In addition, we observed an increased presence of IGKV3-20 (24.8%) in post-M cells. The heavy chain third complementarity-determining region (HCDR3) amino acid lengths of pre-N (average ~17), pre-M (~16), and post-M (~15) cells were commonly distributed and showed no discrepancy. The LCDR3 lengths in pre-N, pre-M, and post-M cells are comparable (average ~9, **Figure S2B**). The antibody genes identified in pre-N cells exhibited germ-like properties in both the variable heavy (V_H_) and light (V_L_) chains (100% and 99.7% median identity to the germline, respectively). However, the sequences obtained from pre-M and post-M cells exhibited more SHMs, with a median of 93.4% and 94.2% identity to the germline for V_H_ and 95.2% and 96.9% for V_L_ (**Figure S2C**), consistent with the possible maturation of pre-M cells in response to prior HCoV infection (8, 26, 29–31).

Next, we cloned and recombinantly expressed 49 immunoglobulin G (IgG) mAbs, 33 of which were derived from pre-N cells, 11 from pre-M cells, and 16 from post-M cells (**Figure 2A**, **Figure S4A**). We identified 19 mAbs from multiple donors that bound to the SARS-CoV-2 S protein, which were used further evaluated (**Figure S3**, **Figure S4A**). Among these mAbs, 4 were derived from pre-N cells, 5 from pre-M cells, and 10 from post-M cells. The pre-N cell-derived mAbs (4/33, 12.12%) bound SARS-CoV-2 with a substantially lower positive rate and binding activity than the pre-M cell (5/11, 45.5%) and post-M cell-derived mAbs (10/16, 62.5%), consistent with the fact that they were derived from naïve B cells that had never encountered SARS-CoV-2 antigen (**Figure 2B**, **Figure S4A**). We further defined the targeted domain of these SARS-CoV-2-binding mAbs by ELISA using the subunits of the SARS-CoV-2 S protein. Among the 4 pre-N cell-derived mAbs, 3 targeted the S2 subunit, and 1 targeted a non-RBD/NTD region on the S1 domain. Among the 5 pre-M cell-derived mAbs, 3 targeted the S2 subunit, and 2 targeted the undetermined domain on S protein. While among the 10 post-M cell-derived mAbs, 5 targeted the RBD, 2 targeted the S2 subunit, and the remaining 3 mAbs targeted the NTD, a non-RBD/NTD region on the S1 domain and the undetermined domain on S protein (**Figure 2C**, **Figure S4B**). Previous reports have found that the B-cell response in the late stages of vaccination showed amplification of antibodies targeting the S1 domain. In contrast, the early stages of the response to the vaccine mainly included the recall of antibodies targeting the S2 domain (32).

We then compared the sequence characteristics of the SARS-CoV-2-binding mAbs from pre-N cells and post-M cells. Compared with the mAbs from post-M cells, the mAbs from pre-N cells exhibited a higher degree of V_H_ germline identity (**Figure 2E**). However, no discrepancy in V_L_ germline identity or CDR3 length was observed (**Figure 2D**). Among the 4 mAbs from the pre-N cells, 3 mAbs employed the IGHV4-34 gene, while no propensity for V-gene pairing was evident among them (**Table 1**). However, mAbs from post-M cells exhibited diversity in V gene usage (**Table 1**).

**Table 1:** Targeting domain and antibody variable region sequence information of SARS-CoV-2 binding mAbs.

Source	mAb	Targeting domain	Heavy chain			Light chain		
			V gene	CDR3 length (aa)	Identity (%)	V gene	CDR3 length (aa)	Identity (%)
pre-N	VA021-44	S1n	IGHV1-46	16	98.3	IGKV4-1	9	97.7
	VA046-16	S2	IGHV4-34	16	100	IGKV3-15	9	100
	VA088-42	S2	IGHV4-34	20	98	IGKV1-5	9	98.2
	VA091-43	S2	IGHV4-34	21	100	IGLV3-21	10	99.7
post-M	VC041-50	RBD	IGHV1-46	16	97.7	IGKV3-15	8	99.6
	VC043-73	RBD	IGHV3-33	16	97	IGKV1-39	9	96.5
	VC043-77	S1n	IGHV3-30	18	95.3	IGKV3-20	9	95.8
	VC088-54	RBD	IGHV1-46	15	99.7	IGKV3-11	10	99.6
	VC088-67	N.D.	IGHV1-69	18	99	IGKV3-20	9	99.7
	VC091-62	RBD	IGHV3-48	15	94.6	IGKV3-20	10	97.2
	VC091-72	RBD	IGHV3-64	15	89.2	IGKV3-15	9	98.6
	VC110-60	S2	IGHV3-21	16	90.5	IGKV3-11	11	95.5
	VC113-60	S2	IGHV3-30	14	98	IGKV4-1	8	97.7
	VC113-67	NTD	IGHV4-39	20	96.3	IGLV1-40	11	98.7

A previous study has shown that germline antibodies are more likely to exhibit polyreactivity compared to mature antibodies with high levels of SHM, and a study of SARS-CoV-2-reactive naïve B cells revealed the presence of polyreactivity in these cells that may be involved in the physiopathology of COVID-19 (25, 33). We then further assessed the polyreactivity of SARS-CoV-2-binding naïve mAbs, no obvious polyreactivity was observed (Data not show).

### mAbs isolated from naïve B cells exhibit a broad spectrum of binding to SARS-CoV-2 and its variants but lack neutralization activity

**Figure 3:**
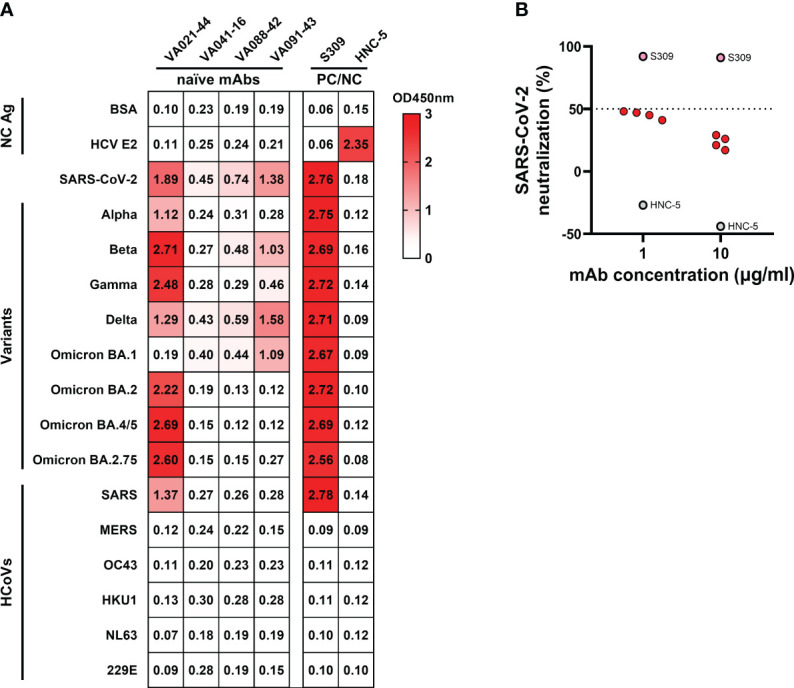
Cross-binding and neutralizing activities of naïve B cell-derived mAbs **(A)** Cross-binding heatmap of naïve B cell-derived mAbs with the S protein of HCoVs and SARS-CoV-2 VOCs, with the mAb S309 and HNC-5 as positive and negative controls, respectively. Colours and numbers represent OD450 nm values for antibody binding to the corresponding S. PC/NC, positive control/negative control. **(B)** Neutralization of SARS-CoV-2 pseudotyped virus by naïve B cell-derived mAbs at two concentrations. The cut-off value was defined as percent neutralization = 50%, and samples with a percent neutralization > 50% were considered to have a neutralizing effect.

Whether ancestral SARS-CoV-2 S-reactive naïve B cells prior to antigen exposure possess binding reactivity to the newly emerging variants remains to be further characterized. Therefore, we tested the binding reactivities of SARS-CoV-2 S-reactive mAbs derived from naïve B cells to the S of circulating viral variants, including Alpha (B.1.1.7), Beta (B.1.351), Gamma (P.1), Delta (B.1.617.2), and Omicron subvariants [BA.1 (B.1.1.529.1), BA.2 (B.1.1.529.2), BA.3 (B.1.1.529.3), and BA.4/5 (B.1.1.529.4/5)]. We also evaluated their binding to the S of other 6 HCoVs in circulation prior to SARS-CoV-2 emergence: SARS-CoV, MERS-CoV, HCoV-OC43, HCoV-HKU1, HCoV-NL63, and HCoV-229E. The results revealed that the 4 anti-SARS-CoV-2 mAbs showed broad-spectrum reactivity to at least 2 variants of SARS-CoV-2. VA021-44, the only mAb targeting the S1 domain, showed the best reactivity against all tested variants except for Omicron BA.1 (**Figure 3A**). In addition, VA021-44 also cross-bound to SARS-CoV, similar to the binding profile of the broad-spectrum nAb S309, although VA021-44 typically exhibited lower binding activity. However, none of the mAbs exhibited cross-reactivity to MERS-CoV or the 4 endemic HCoVs tested. We further tested the binding reactivities of SARS-CoV-2 S-reactive mAbs derived from preexisting memory B cells to these S proteins. And 3 showed broad-spectrum reactivity to most of the variants of SARS-CoV-2 (**Figure S4C**). Among them, 2 mAbs exhibited cross-binding to all 4 endemic HCoVs and other HCoVs. The results indicate that the naïve and preexisting memory mAbs exhibited binding to primarily SARS-CoV-2 and its variants, but cross-reactivity of the naïve mAbs was detected only for SARS-CoV but not to MERS-CoV or other endemic HCoVs. The lack of binding to other HCoVs suggests that isolated SARS-CoV-2-reactive naïve B cells are more likely to have originated from the SARS-CoV-2-reactive naïve repertoire rather than have been induced by prior HCoV infection. Finally, using pseudotyped SARS-CoV-2 S virus, we performed a neutralization analysis of the SARS-CoV-2-binding mAbs derived from naïve B cells. Unfortunately, the results showed that none of the 4 mAbs displayed potent neutralizing activity (**Figure 3B**).

### Evolutionary analysis of IGHV4-4/IGKV3-20 lineage mAbs in a single donor

**Figure 4:**
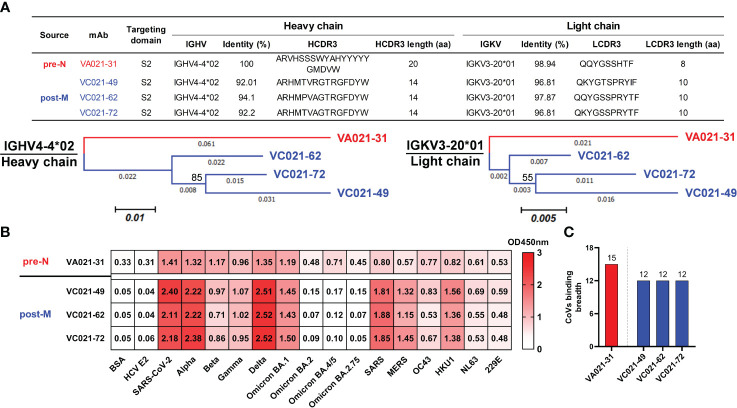
Tracking IGHV4-4/IGKV3-20 lineage mAb maturation in a single donor after vaccination **(A)** Targeting domain and variable region sequence information (top) and molecular phylogenetic analysis (bottom) performed by the maximum likelihood method for the IGHV4-4/IGKV3-20 mAbs from donor V021. The tree with the highest log likelihood is shown and drawn to scale, with branch lengths measured in the number of substitutions per site. The percentage of trees in which the associated taxa clustered together is shown next to the branches. The evolutionary history of the taxa was evaluated by the bootstrap method inferred from 1000 replicates. **(B)** Cross-binding heatmap of the IGHV4-4/IGKV3-20 mAbs to HCoV S and SARS-CoV-2 VOC S proteins. Colours and numbers represent OD450 nm values for antibody binding to the corresponding S. **(C)** The binding breadth of the IGHV4-4/IGKV3-20 mAbs to the assayed CoV S proteins. The number on the bar represents the number of each mAb binding to the tested CoVs.

To further understand the evolutionary characteristics of these SARS-CoV-2-reactive naïve B cells after antigenic stimulation, we analysed the V genes used by mAbs isolated from a single donor. Interestingly, we found that a class of mAbs using the same IGHV4-4/IGKV3-20 gene was present at both pre- and post-vaccination and that all of these mAbs bound SARS-CoV-2 S. A pre-N cell-derived mAb (VA021-31) and 3 corresponding mature post-M cell-derived mAbs (VC021-49, VC021-62, and VC021-72) isolated from donor V021 that used identical IGHV4-4 and IGKV3-20 genes were then selected and cloned for evolutionary analysis (**Figure 4A**, top). All 4 cloned IGHV4-4/IGKV3-20 mAbs targeted the S2 domain. These mAbs also shared similar CDR3 amino acid sequences, especially the 3 mature post-M cell-derived mAbs, which had only 2 and 3 amino acid differences in HCDR3 and LCDR3, respectively. Compared to VA021-31, the mature mAbs isolated from post-M cells had accumulated more SHMs as a result of being more mature after antigenic stimulation. The mature mAbs all bear equal-length HCDR3 and LCDR3, with a shorter HCDR3 and a slightly longer LCDR3 than VA021-31 (**Figure 4A**, top). We inferred their sequence-based evolutionary characteristics by constructing a molecular phylogenetic evolutionary tree analysis of such mAbs. The bootstrap values were 85% and 55% for VH and VL, respectively (**Figure 4A**, bottom).

We then evaluated the binding activity of these IGHV4-4/IGKV3-20-derived mAbs to ancestral SARS-CoV-2 and its variants, as well as other preexisiting HCoVs, by ELISA. VA021-31 engaged all 15 CoVs we tested, which showed stronger binding to S of ancestral SARS-CoV-2 and several variants. Regarding mature VC021-49, VC021-62, and VC021-72, their binding activities to multiple SARS-CoV-2 variants and HCoVs were enhanced, but their binding activities to several newly emerging variants were lost (**Figure 4B**). This result seems to indicate that VA021-31 lineage maturation upon vaccination promoted convergent evolution under vaccine antigen stimulation (**Figure 4C**). Collectively, these data indicate that SARS-CoV-2-reactive naïve B cells, after antigenic activation, were able to undergo SHM and thus enhance their binding activity to multiple related viruses, but there was a reduction in their binding breadth. Although rather weak, we observed broad cross-reactivity of these IGHV4-4/IGKV3-20-derived mAbs to all preexisting HCoVs. This finding may suggest that such a naïve B-cell germline is a broad precursor commonly shared by HcoVs.

## Discussion

Previous studies have identified SARS-CoV-2 S-reactive naïve B cells and preexisting memory B cells in unexposed individuals (22, 25, 26, 34). Whether and how these S-reactive B cells contribute to vaccine-elicited antibody responses has not been defined. Here, we demonstrated that approximately 0.091% of naïve B cells are reactive to the SARS-CoV-2 S protein and that the frequency of S-reactive naïve B cells before vaccination was positively associated with the frequency of S-reactive memory B cells after vaccination. There is also some evidence showing that SARS-CoV-2 infection significantly boosts endemic CoV-specific antibody responses, although these antibodies are less associated with protection (9, 27, 29). We demonstrated a positive correlation between S-reactive memory B cells before vaccination and S-reactive memory B cells after vaccination. Preexisting S-reactive memory B cells, generated by endemic CoV infection, may be engaged simultaneously as well as S-reactive naïve B cells after vaccination (8, 32, 35). Thus, both S-reactive naïve B cells and preexisting memory B cells may act as major cocontributing sources of vaccine-elicited antibody responses.

The BCRs of the SARS-CoV-2 S-reactive naïve B cells we characterized show germline-like properties with low levels of SHM, consistent with early reports confirming the presence of a large proportion of anti-SARS-CoV-2 precursor naïve B cells in unexposed individuals (22, 25). Isolated mAbs from S-reactive naïve B cells exhibited low binding capacities to S protein compared with those of mAbs from S-reactive memory B cells before and after vaccination. Antibody Target mapping revealed that the reactive naïve B cell-derived mAbs here mainly target non-immunodominant S2 subunit. Since the S2 of CoVs is typically more conserved, epitopes on S2 may be more prevalent in many CoVs, which is formative for broader-spectrum CoVs vaccine design. Unfortunately, none of these naïve mAbs we isolated from small sample size neutralized the pseudotyped SARS-CoV-2 S virus. Although antibody protection mainly relies on the neutralization activity, non-neutralizing antibodies antibodies have also been shown to be protective in many viral infections via Fc effect (36–40). Therefore, Fc effect of these naive B cell-derived mAbs remain to be further investigated to confirm their contribution in protection.

Moreover, these naïve B cell-derived mAbs show broad binding profiles to SARS-CoV-2 and its variants, revealing their potential to mature into broadly active antibodies after antigen exposure. It has been reported that SARS-CoV-2 infection can drive the affinity maturation of these potential B-cell precursors and the induction of protective immunity (4, 16, 29). And the cross-reactivity to endemic HCoVs of mAbs derived from preexisting memory B cells may partially explain where may these cells arise from, and further studies should assess the contribution of SARS-CoV-2 preexisting memory B cells to protective antibody response (9, 41). By tracking IGHV4-4/IGKV3-20 lineage evolution before and after vaccination in a single donor, we confirmed that germline-like precursor BCRs underwent affinity maturation with increased SHM levels and enhanced binding capacities to SARS-CoV-2 and multiple related CoV S proteins after vaccination.

Current vaccines efficiently elicit robust nAb responses, but the production of large amounts of nonprotective antibodies is also induced (8). High frequencies of SARS-CoV-2 S-reactive naïve B cells exist in unexposed individuals, and these germ-like BCRs can be induced and matured by vaccination, providing the possibility to precisely direct specific protective precursor BCR responses by optimized antigens. This concept has been successful in HIV vaccine design (18–21). Thus, precisely targeting S-reactive naïve B cells may be a high-priority strategy for next-generation SARS-CoV-2 vaccine development. Several limitations, such as the small sample size and low number of antibodies isolated from S-reactive naïve B cells in this study, limit us from obtaining more information about naïve B cells. Exploring whole S-reactive naïve BCR repertoires and tracking the maturation pathway driven by optimized antigens would be beneficial for BCR-based vaccine design (21, 42–44).

In conclusion, our study primarily revealed that S-reactive naïve B cells and preexisting memory B cells cocontribute to antibody responses after vaccination. Targeting the BCRs of S-reactive naïve B cells would help guide precise design of SARS-CoV-2 vaccines.

## Materials and methods

### Study subjects and sample collection

A total of 11 donors who had received two doses of SARS-CoV-2 inactivated vaccines (Sinovac, Beijing, China) were recruited from the First People’s Hospital of Chenzhou, Hunan Province, China (**Supplementary Table 1**). None of the vaccinees had subsequent SARS-CoV-2 or variant exposure, as determined by frequent PCR testing and questionnaire administration during the observation period. Peripheral blood samples from the donors were collected before vaccination (day 0) and 14 days after the second vaccine dose (approximately equivalent to 45 days after the first dose). Each participant in this study had reached the age of 18 and was free from any underlying illnesses. PBMCs and plasma were isolated and then frozen in liquid nitrogen and a −80°C freezer, respectively. Each participant signed a written consent form. The study protocol was approved by the First People’s Hospital of Chenzhou (V.3.0, 2021001).

### Single B-cell sorting

SARS-CoV-2 S labelled with Alexa Fluor 488 and Alexa Fluor 647 (Thermo Fisher Scientific, Waltham, MA, USA), as probes, was used to identify SARS-CoV-2-reactive B cells. PBMCs in liquid nitrogen were rapidly thawed in a 37°C water bath before being incubated overnight in RPMI 1640 medium containing 10% foetal bovine serum (FBS) in a 37°C, 5% CO_2_ incubator (Thermo Fisher Scientific, Waltham, MA, USA). Following two washes with staining buffer (2% FBS and 1% HEPES in PBS), PBMCs were stained for 30 min with the LIVE/DEAD Fixable Blue Dead Cell Stain Kit (Thermo Fisher Scientific, Waltham, MA, USA) and then treated with an anti-ACE2 antibody and rabbit polyclonal antibody (Sino Biological, Beijing, China) to block the S-ACE2 binding site. After being washed, PBMCs were stained with the SARS-CoV-2 S probe mixture for 30 min at 4°C while avoiding light. PBMCs were washed two times to remove excess reagents. The treated PBMCs were stained with antibodies that had been pretitrated to an optimized dilution and fluorescently labelled in 96-well V-bottom plates at 4°C for 30 min. The fluorescently labelled antibodies were as follows: BUV737 mouse anti-human CD3 (SK7), PE mouse anti-human IgG (G18-145) (BD Biosciences, Franklin Lake, NJ, USA), PerCP/Cyanine5.5 mouse anti-human CD19 (HIB19), PE-cy7 mouse anti-human CD27 (M-T271), and PE/Dazzle 594 mouse anti-human IgD (IA6-2) (BioLegend, San Diego, CA, USA). After washing, single SARS-CoV-2 S-reactive naïve B cells (CD3^-^CD19^+^CD27^-^IgD^+^IgG^-^ SARS-CoV-2 S-Alexa Fluor 488^+^ SARS-CoV-2 S-Alexa Fluor 647^+^) and single memory B cells (CD3^-^CD19^+^CD27^+^IgD^-^IgG^+^ SARS-CoV-2 S-Alexa Fluor 488^+^ SARS-CoV-2 S-Alexa Fluor 647^+^) were sorted using a MoFlo XDP (Beckman Coulter, Brea, CA, USA) flow cytometer into a 96-well PCR plate (Thermo Fisher Scientific, Waltham, MA, USA) containing lysis buffer (10×PBS, 5 mM DTT, 1% IGEPAL, 20 ng/μl random primer, 2 U/μl RNasin Ribonuclease Inhibitor), which was placed at -80°C before subsequent processing. Flow cytometry data were analysed using FlowJo software version 10.8.1 (Tree Star, San Carlos, CA, USA).

### Antibody variable gene amplification and analysis

Antibody variable gene mRNA transcripts, VH, variable kappa (Vκ), and variable lambda (Vλ), were amplified by RT−PCR in a previously reported manner (45). In brief, cDNA was synthesized using SuperScript IV Reverse Transcriptase (Thermo Fisher Scientific, Waltham, MA, USA), followed by two rounds of nested PCR. The PCR products were purified and sequenced to determine the heavy and kappa/lambda chain sequences, and the variable region sequences of the heavy and light chains were synthesized and inserted separately into Ig heavy- or light-chain expression vectors: AbVec2.0-IGHG1, AbVec1.1-IGKC, and AbVec1.1-IGLC2 (Addgene, Watertown, MA, USA). IgBLAST and IMGT/V-QUEST were used to assess antibody CDR3 length, variable region gene usage, and mutation information.

### Expression and purification of IgG mAbs

To express recombinant mAbs, heavy chain and matched light chain antibody expression vectors (0.5 μg/mL) were chemically cotransfected at a 1:2 ratio into 2×10^6^ cells/ml FreeStyle 293-F cells using 8 μg/mL polyethyleneimine (Polysciences, USA) transfection reagent. Transfected cells were propagated at 37°C and in 8% CO_2_ under constant shaking at 105 rpm in serum-free FreeStyle 293 Expression Medium (Thermo Fisher Scientific, Waltham, MA, USA) for 5-7 days. Cell supernatants were harvested by centrifugation at 4,000 × g and filtered through a 0.22 μm filter after culture. The suspension was purified using HiTrap Protein A columns HP (Cytiva, Marlborough, MA, USA) with an ÄKTA pure system (Cytiva, Marlborough, MA, USA), washed with PBS, and then eluted with glycine (pH 3.0) into a collection tube containing Tris-HCl buffer (pH 8.0). Buffer exchange was accomplished by dialyzing three times in PBS, and the final concentration of purified antibodies was determined using a Pierce BCA Protein Assay Kit (Thermo Fisher Scientific, Waltham, MA, USA). Purified antibodies were then kept at -40°C until further use.

### Enzyme-linked immunosorbent assay

S proteins and S subunits used in the ELISA were obtained commercially (Sino Biological, Beijing, China), including: 1) the S proteins of HCoVs: SARS-CoV-2, SRAS-CoV, MERS-CoV, HCoV-OC43, HCoV-HKU1, HCoV-NL63 and HCoV-229E (40589-V08B1, 40634-V08B, 40069-V08B, 40607-V08B, 40606-V08B, 40604-V08B, and 40605-V08B, respectively); 2) the S proteins of SARS-CoV-2 variants: Alpha (B.1.1.7, 40589-V08B6), Beta (B.1.351, 40589-V08B11), Gamma (P.1, 40589-V08B10), Delta (B.1.617.2, 40589-V08B16), and Omicron subvariants BA.1 (B.1.1.529.1, 40589-V08B33), BA.2 (B.1.1.529.2, 40589-V08H28), BA.4/5 (B.1.1.529.4/5, 40589-V08H32) and BA.2.75 (B.1.1.529.2.75, 40589-V08H36); and 3) the SARS-CoV-2 S1, S2, RBD and NTD subunits (40591-V08H, 40590-V08B, 40592-V08B and 40591-V49H, respectively). The reactivity of mAbs to S proteins or subunits of HCoVs or variants was assayed by ELISA. Briefly, 96-well ELISA plates (Corning, NY, USA) were coated with S proteins of corresponding CoVs or SARS-CoV-2 S subunits (2 μg/ml) in PBS at 100 μl/well and incubated overnight at 4°C. The plates were washed five times with PBS containing 0.5% Tween-20 (PBS-T solution) and blocked with 200 μl of blocking buffer per well (PBS containing 2% FBS and 2% bovine serum albumin (BSA) (Sigma-Aldrich, St. Louis, MO, USA)) for 2-4 hours at room temperature (RT). The plates were washed five times with PBS-T, and 100 μl of isolated mAbs (10 μg/ml) per well in blocking buffer were added to the wells and incubated for 1 hour at 37°C. The plates were washed five times with PBS-T, and then 100 μl per well Peroxidase-AffiniPure goat anti-human IgG (H+L) antibody (Jackson ImmunoResearch, PA, USA) was added at a 1:5,000 dilution in blocking buffer for 1 hour at 37°C. The plates were washed five times with PBS-T and developed with 100 μl per well 3,3’,5,5’-tetramethylbenzidine (TMB) substrate (Thermo Fisher Scientific, Waltham, MA, USA) for 5 min at RT. The reaction was terminated by addition of 50 μl per well 1 M H_2_SO_4_. Absorbance was measured using an RT-6100 (Rayto, Shenzhen, China) enzyme analyser at 450 nm. All ELISA experiments were performed in duplicate, and the data were averaged over three independent experiments. Positive binding of a mAb was defined by OD450 nm (optical density at 450 nm) ≥ 0.40. BSA and an unrelated hepatitis C virus (HCV) antigen were used as negative antigen controls for the ELISA binding test. An approved anti-CoV broad-spectrum neutralizing monoclonal antibody, S309 (46), was used as a positive control, and an unrelated anti-HCV antibody HNC-5 was used as a negative control.

### Pseudotyped particle neutralization assay

SARS-CoV-2 neutralization by the mAbs was measured by a reduction in luciferase activity after infection of ACE2-expressing 293T cells (ACE2-293T) with the S pseudovirus, as previously described (47, 48). Briefly, ACE2-293T cells were seeded in a 96-well plate (Corning, NY, USA) (2×10^4^ cells/well) and cultivated for 24 hours at 37°C with 5% CO_2_. Before the experiment, 165 μl/well of diluted mAbs (1 μg/ml and 10 μg/ml) in DMEM with 50 μg/mL streptomycin was applied in triplicate to a 96-well plate. Then, 75 μl of pseudovirus diluted to 10,000 TCID_50_/ml was added and incubated at 37°C for 1 hour. After cell incubation, the supernatant was removed, and 70 μl of the virus-antibody mixture was added and incubated at 37°C for 24 hours before being replenished at 100 μl/well and incubated for another 24 hours. After discarding the supernatant, 50 μl of cell lysis buffer was added and incubated at RT for 30 minutes on a shaking table, 30 μl of which was then transferred to an optical white bottom plate. Each well received 50 μl of luciferase reagent to measure luciferase activity using relative light units (RLU). Negative (VC, virus+cells) and blank (NC, cells) controls were included in parallel. The percentage of neutralization was calculated by the following equation:


$$\%\;Neutralization=100\%*\left[1-\frac{RLU\left(tested\;samples\right)}{RLU\left(VC\right)+RLU\left(NC\right)}\right]$$

Positive neutralization of mAbs was defined by ≥50% neutralization of the pseudovirus. S309 and HNC-5 were the positive and negative controls, respectively.

## Data availability statement

The original contributions presented in the study are included in the article/**Supplementary Materials**. Further inquiries can be directed to the corresponding authors.

## Ethics statement

The studies involving humans were approved by the First People’s Hospital of Chenzhou (V.3.0, 2021001). The studies were conducted in accordance with the local legislation and institutional requirements. The participants provided their written informed consent to participate in this study.

## Author contributions

ST: Conceptualization, Data curation, Methodology, Software, Visualization, Writing – original draft, Writing – review & editing. YH: Conceptualization, Funding acquisition, Methodology, Resources, Writing – review & editing. YW: Data curation, Methodology, Validation, Writing – review & editing. YT: Data curation, Validation, Writing – review & editing. QW: Methodology, Validation, Writing – review & editing. XZ: Validation, Writing – review & editing. RL: Validation, Writing – review & editing. DP: Validation, Writing – review & editing. FL: Validation, Writing – review & editing. TX: Validation, Writing – review & editing. CW: Validation, Writing – review & editing. YL: Methodology, Writing – review & editing. WL: Conceptualization, Funding acquisition, Resources, Supervision, Writing – review & editing. XQ: Conceptualization, Funding acquisition, Methodology, Resources, Supervision, Writing – review & editing, Writing – original draft.

## Supplementary material

Supplementary Figure 1 Single SARS-CoV-2 spike-reactive B-cell sorting strategy Representative FACS gating strategy for PBMCs from donor V032. Gating was performed on naïve (CD3^-^CD19^+^CD27^-^IgD^+^IgG^-^) and memory (CD3^-^CD19^+^CD27^+^IgD^-^IgG^+^) B cells pre- and post-vaccination. Both Alexa Fluor 488- and Alexa Fluor 647-labelled S proteins were probes used to define S-reactive B cells. Sort gating is denoted by the black arrow. Gating was based on mean fluorescence intensity “minus one” (FMO) control and the FMO of SARS-CoV-2 spike Alexa Fluor 488 and Alexa Fluor 647 are shown on the right of the figure.

Supplementary Figure 2 Sequence analysis of mAbs isolated from SARS-CoV-2 spike-reactive B cells **(A)** Heatmap of V_H_ and V_L_ gene usage of acquired antibody sequences derived from pre-N (n=48), pre-M (n=20), and post-M (n=37) cells. Colours represent the percent of total sequences for each cell group. HCDR3 and LCDR3 amino acid (aa) length **(B)** and identity to inferred germline gene sequences of V_H_ and V_L_ **(C)** of isolated pre-N, pre-M, and post-M antibody sequences. Data are presented as the mean values and median percent values for B and C, respectively. The differences among multiple groups for CDR3 length and identity to germline were evaluated by ordinary one-way ANOVA and the Kruskal−Wallis test, respectively. The differences within groups were evaluated by Tukey’s multiple comparisons test and Dunn’s multiple comparisons test. *****p*< 0.0001. *p<* 0.05 was considered to indicate a two-tailed significant difference, ns, not significant.

Supplementary Figure 3 Primary screening of isolated mAb binding with the SARS-CoV-2 spike protein The scatter plot shows mAbs binding to SARS-CoV-2 S. S309 (pink) and HNC-5 (grey) were used as positive controls for SARS-CoV-2 S and HCV E2 binding, respectively. The cut-off value was defined as OD450 nm = 0.4, and samples with an OD450 nm > 0.4 were considered to be positive for binding.

Supplementary Figure 4 Characterization of SARS-CoV-2 S-reactive preexisting memory B cell-derived mAbs **(A)** The preexisting memory B cell-derived mAbs binding to SARS-CoV-2 S. S309 (pink) and HNC-5 (grey) were used as positive controls for SARS-CoV-2 S and HCV E2 binding, respectively. The numbers and percentages above indicate the proportion of SARS-CoV-2 S-binding mAbs. The cut-off value was defined as OD450 nm = 0.4, and mAbs with an OD450 nm > 0.4 were considered positive for binding. **(B)** Distribution of preexisting memory B cell-derived mAbs targeting domains in the S of SARS-CoV-2. S2, S2 subunit. S1n, S1 non-RBD/NTD domain. RBD, RBD. NTD, NTD. N.D., not detected. **(C)** Cross-binding heatmap of preexisting memory B cell-derived mAbs with the S protein of HCoVs and SARS-CoV-2 VOCs, with the mAb S309 and HNC-5 as positive and negative controls, respectively. Colours and numbers represent OD450 nm values for antibody binding to the corresponding S. PC/NC, positive control/negative control.
