# Supplementary material for: SARS-CoV-2 spike-reactive naïve B cells and pre-existing memory B cells contribute to antibody responses in unexposed individuals after vaccination
Source: Front Immunol. 2024 Feb 14;15:1355949. doi: 10.3389/fimmu.2024.1355949 (PMC10899457; doi:10.3389/fimmu.2024.1355949)
Supplement: Supplementary file 1 [file Table_1.docx]

**Table S1. Basic information about the vaccine recipients recruited for this study.**

| **Vaccine recipient** | **Age (years)** | **Sex** | **PBMCs availability** | |
| --- | --- | --- | --- | --- |
|  |  |  | **Before first dose vaccine (days)** | **After second dose vaccine (days)** |
| Vaccine recipient 1 | 25 | Male | 0 | 17 |
| Vaccine recipient 2 | 42 | Female | 0 | 16 |
| Vaccine recipient 3 | 34 | Female | 0 | 19 |
| Vaccine recipient 4 | 44 | Male | 0 | 17 |
| Vaccine recipient 5 | 45 | Female | 0 | 17 |
| Vaccine recipient 6 | 40 | Female | 0 | 17 |
| Vaccine recipient 7 | 43 | Female | 0 | 17 |
| Vaccine recipient 8 | 33 | Female | 0 | 16 |
| Vaccine recipient 9 | 45 | Female | 0 | 17 |
| Vaccine recipient 10 | 35 | Male | 0 | 17 |
| Vaccine recipient 11 | 24 | Female | 0 | 13 |
| Median | 40 |  | 0 | 17 |
| (IQR) | (33-44) |  | (0-0) | (16-17) |
