# Supplementary figures and images for: SARS-CoV-2 spike-reactive naïve B cells and pre-existing memory B cells contribute to antibody responses in unexposed individuals after vaccination
Source: Front Immunol. 2024 Feb 14;15:1355949. doi: 10.3389/fimmu.2024.1355949 (PMC10899457; doi:10.3389/fimmu.2024.1355949)

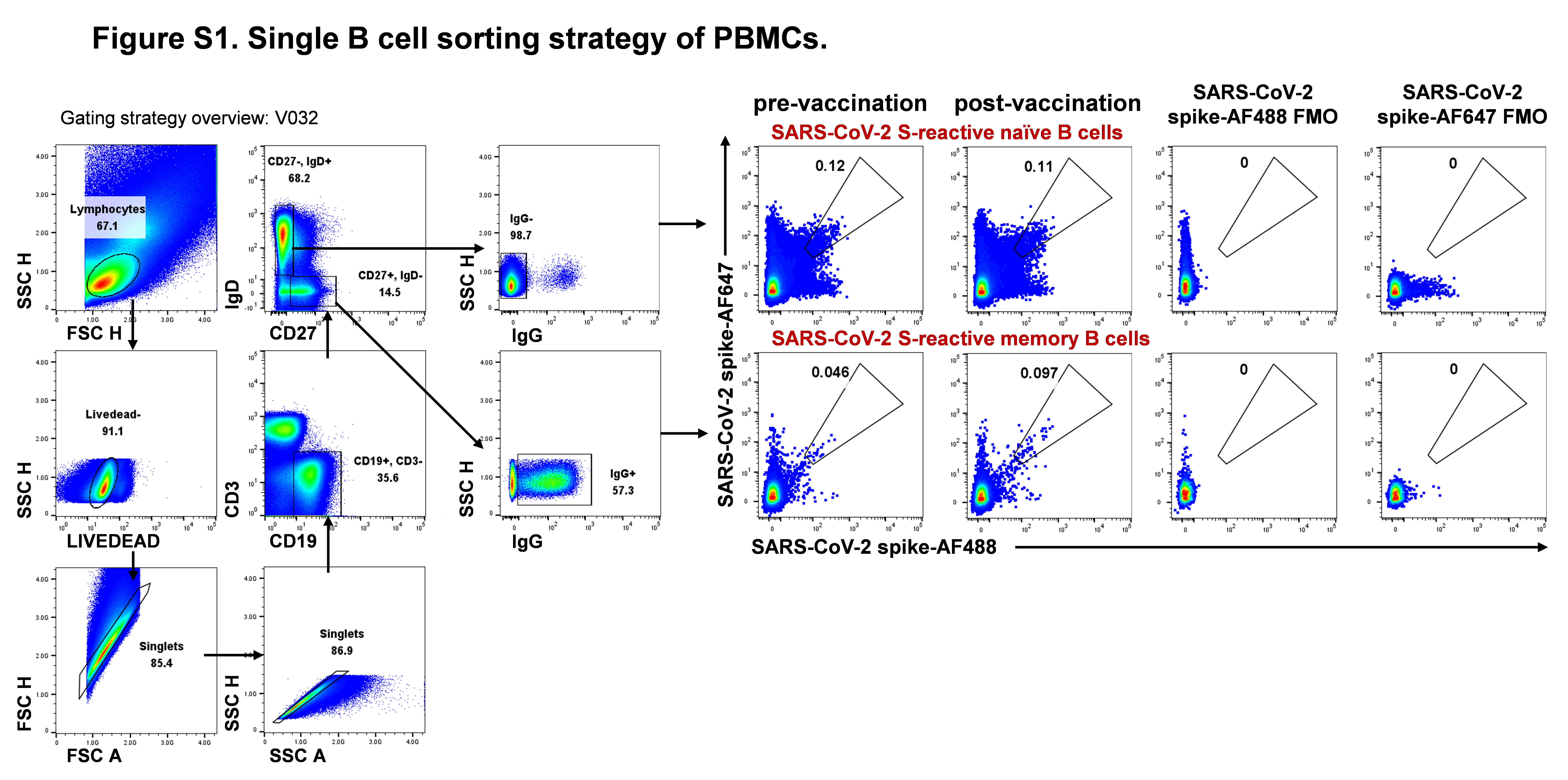

Supplement: Supplementary Figure 1 — Single SARS-CoV-2 spike-reactive B-cell sorting strategy Representative FACS gating strategy for PBMCs from donor V032. Gating was performed on naïve (CD3-CD19+CD27-IgD+IgG-) and memory (CD3-CD19+CD27+IgD-IgG+) B cells pre- and post-vaccination. Both Alexa Fluor 488- and Alexa Fluor 647-labelled S proteins were probes used to define S-reactive B cells. Sort gating is denoted by the black arrow. Gating was based on mean fluorescence intensity “minus one” (FMO) control and the FMO of SARS-CoV-2 spike Alexa Fluor 488 and Alexa Fluor 647 are shown on the right of the figure. [file Image_1.png]

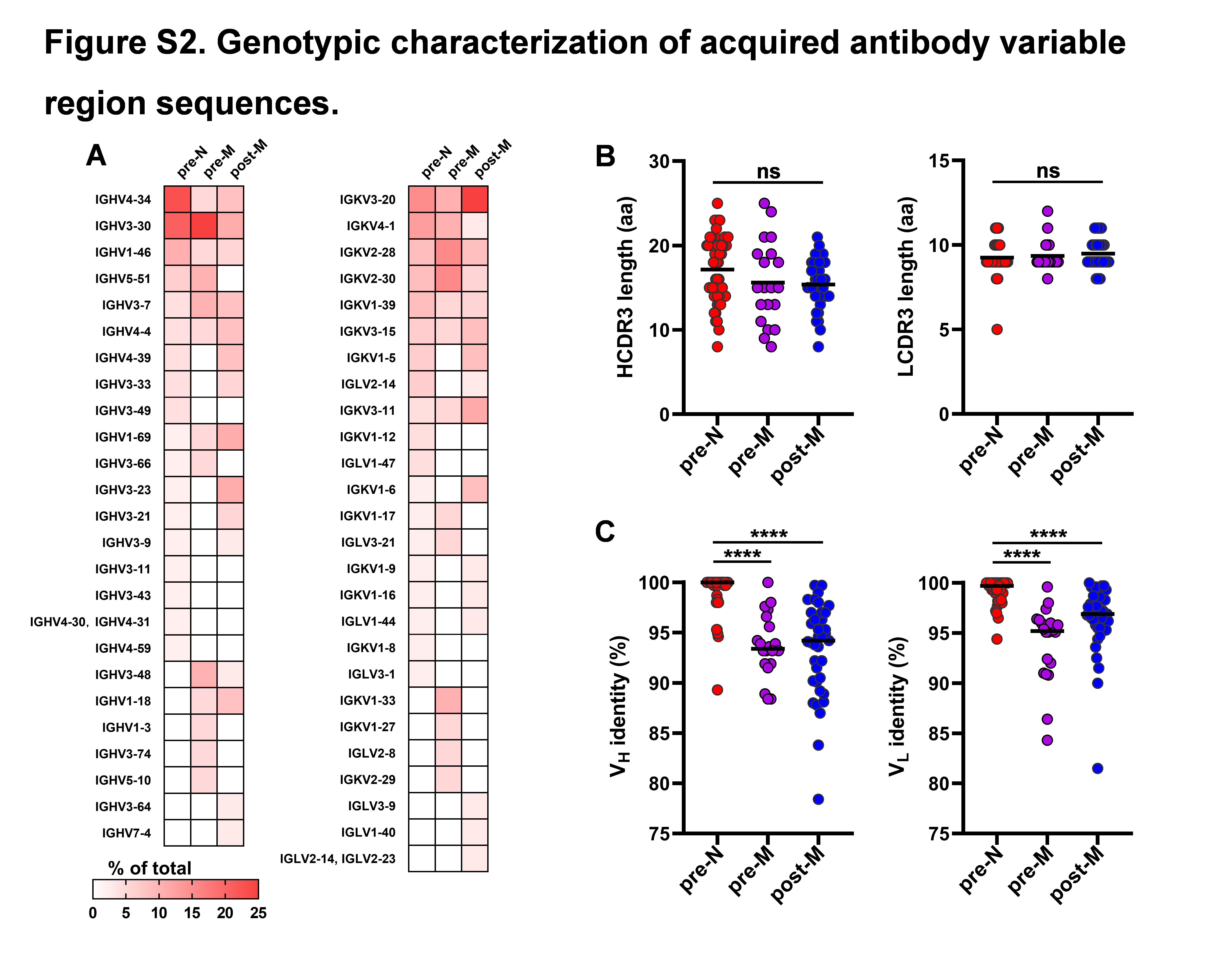

Supplement: Supplementary Figure 2 — Sequence analysis of mAbs isolated from SARS-CoV-2 spike-reactive B cells (A) Heatmap of VH and VL gene usage of acquired antibody sequences derived from pre-N (n=48), pre-M (n=20), and post-M (n=37) cells. Colours represent the percent of total sequences for each cell group. HCDR3 and LCDR3 amino acid (aa) length (B) and identity to inferred germline gene sequences of VH and VL (C) of isolated pre-N, pre-M, and post-M antibody sequences. Data are presented as the mean values and median percent values for B and C, respectively. The differences among multiple groups for CDR3 length and identity to germline were evaluated by ordinary one-way ANOVA and the Kruskal−Wallis test, respectively. The differences within groups were evaluated by Tukey’s multiple comparisons test and Dunn’s multiple comparisons test. ****p< 0.0001. p< 0.05 was considered to indicate a two-tailed significant difference, ns, not significant. [file Image_2.png]

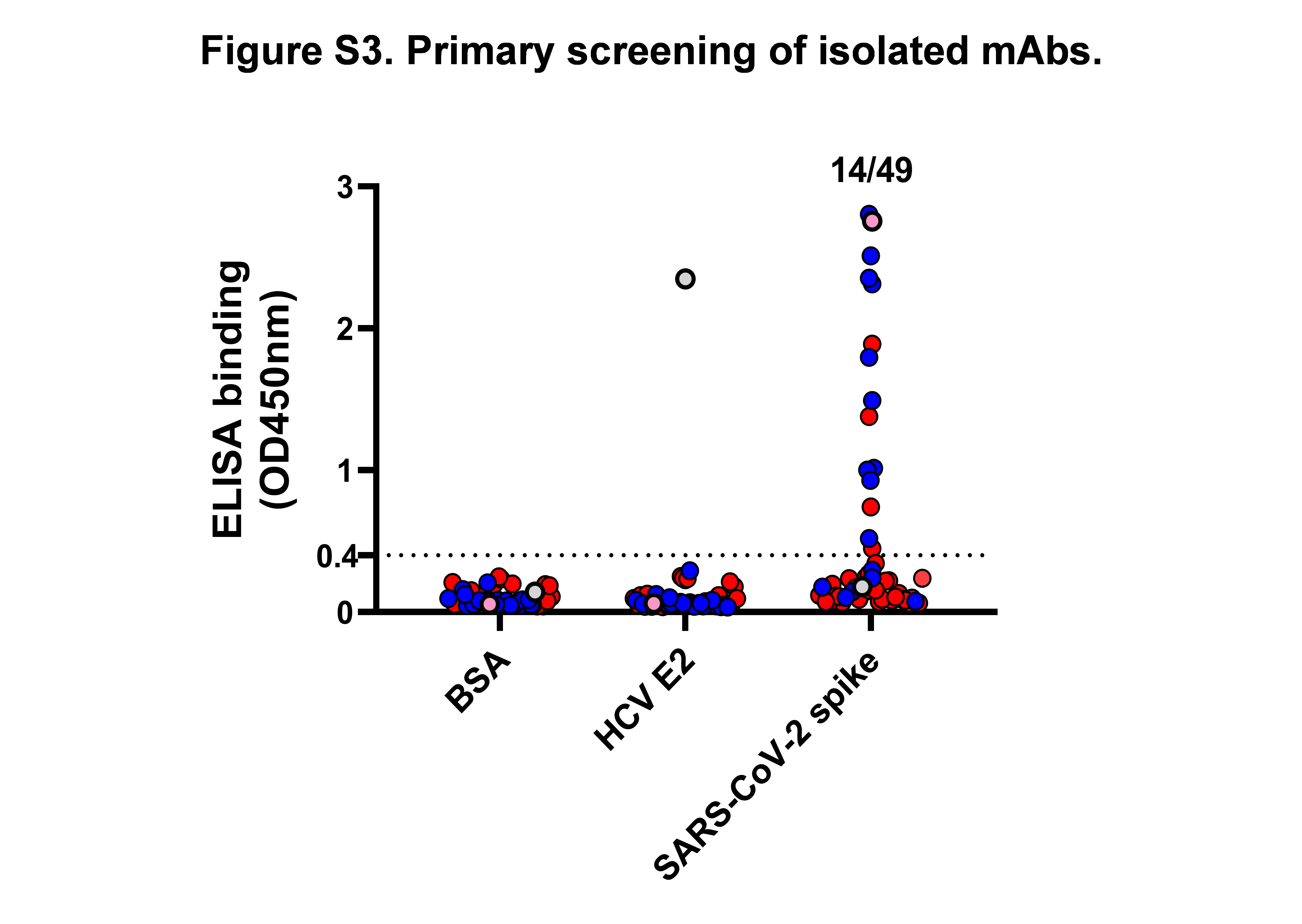

Supplement: Supplementary Figure 3 — Primary screening of isolated mAb binding with the SARS-CoV-2 spike protein The scatter plot shows mAbs binding to SARS-CoV-2 S. S309 (pink) and HNC-5 (grey) were used as positive controls for SARS-CoV-2 S and HCV E2 binding, respectively. The cut-off value was defined as OD450 nm = 0.4, and samples with an OD450 nm > 0.4 were considered to be positive for binding. [file Image_3.png]

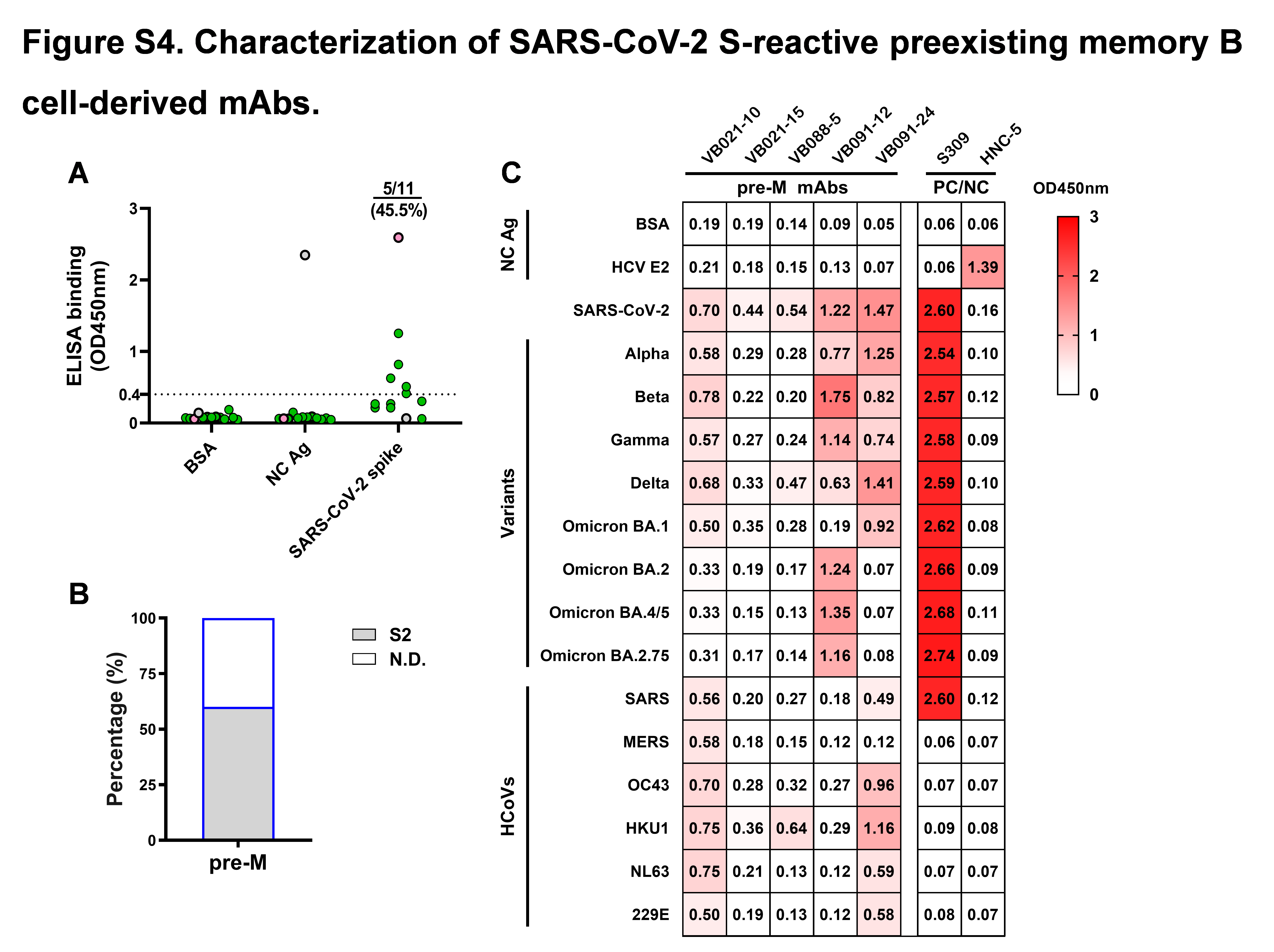

Supplement: Supplementary Figure 4 — Characterization of SARS-CoV-2 S-reactive preexisting memory B cell-derived mAbs (A) The preexisting memory B cell-derived mAbs binding to SARS-CoV-2 S. S309 (pink) and HNC-5 (grey) were used as positive controls for SARS-CoV-2 S and HCV E2 binding, respectively. The numbers and percentages above indicate the proportion of SARS-CoV-2 S-binding mAbs. The cut-off value was defined as OD450 nm = 0.4, and mAbs with an OD450 nm > 0.4 were considered positive for binding. (B) Distribution of preexisting memory B cell-derived mAbs targeting domains in the S of SARS-CoV-2. S2, S2 subunit. S1n, S1 non-RBD/NTD domain. RBD, RBD. NTD, NTD. N.D., not detected. (C) Cross-binding heatmap of preexisting memory B cell-derived mAbs with the S protein of HCoVs and SARS-CoV-2 VOCs, with the mAb S309 and HNC-5 as positive and negative controls, respectively. Colours and numbers represent OD450 nm values for antibody binding to the corresponding S. PC/NC, positive control/negative control. [file Image_4.png]
